# Therapeutic efficacy of *Centratherum anthelminticum* in subclinical mastitis: A biochemical and hematological assessment in lactating cattle

**DOI:** 10.14202/vetworld.2025.1741-1747

**Published:** 2025-06-26

**Authors:** Muhammad Adil, Farrah Deeba, Muhammad Tariq, Muhammad Usman, Saba Saeed, Eliana Ibáñez-Arancibia, Patricio De los Ríos-Escalante, Muhammad Safdar

**Affiliations:** 1 Department of Clinical Medicine and Surgery, University of Agriculture, Faisalabad, Pakistan; 2 College of Animal Science and Technology, Nanjing Agricultural University, Nanjing, Jiangsu, 210095, PR China; 3 Department of Basic Sciences, University of Veterinary and Animal Sciences, Lahore, Sub-Campus Narowal, Pakistan; 4 Department of Zoology, The Government Sadiq College Women University, 63100, Bahawalpur, Punjab, Pakistan; 5 PhD Program in Sciences Mentioning Applied Molecular and Cell Biology, La Frontera University, Temuco, Chile; 6 Laboratory of Engineering, Biotechnology and Applied Biochemistry, Department of Chemical Engineering, Faculty of Engineering and Science, La Frontera University, Temuco, Chile; 7 Department of Biological and Chemical Sciences, Faculty of Natural Resources, Catholic University of Temuco, Temuco, Chile; 8 Department of Biological and Chemical Sciences, Faculty of Natural Resources, Catholic University of Temuco, Temuco, Chile; 9 Nucleus of Environmental Sciences, Faculty of Natural Resources, Catholic University of Temuco, Temuco, Chile; 10 Faculty of Animal Production and Technology, Cholistan University of Veterinary and Animal Sciences, 63100, Bahawalpur, Pakistan

**Keywords:** antioxidant capacity, *Centratherum anthelminticum*, dairy cattle, milk quality, oxidative stress, somatic cell count, subclinical mastitis, tylosin

## Abstract

**Background and Aim:**

Subclinical mastitis (SCM) in dairy cattle significantly compromises milk quality, animal health, and farm profitability, often remaining undetected due to the absence of clinical signs. The increasing antimicrobial resistance associated with conventional treatments highlights the need for effective alternatives. This study aimed to evaluate the therapeutic potential of *Centratherum anthelminticum* (CA), alone and in combination with tylosin, in managing SCM in lactating cows.

**Materials and Methods:**

Fifteen California mastitis test-positive cows were randomly divided into three groups (n = 5/group). Group A received tylosin (18 mg/kg intramuscular), Group B received CA (120 g orally), and Group C received both treatments. Milk samples were analyzed pre- and post-treatment for somatic cell count (SCC), pH, electrical conductivity, fat, protein, lactose, and solids-not-fat (SNF) content. Hematological parameters, including red blood cell (RBC), white blood cell, hemoglobin, packed cell volume (PCV), and lymphocyte percentages, were evaluated alongside oxidative stress markers – total antioxidant capacity (TAC) and total oxidative stress (TOS).

**Results:**

Significant post-treatment reductions in SCC, pH, and conductivity were observed in all groups. Group C exhibited the most pronounced improvements in lactose, fat, and SNF, with no change in protein. Group B demonstrated the highest TAC increase and TOS reduction, affirming CA’s antioxidative potential. Hematological evaluations revealed systemic improvements post-treatment, particularly in RBC and PCV levels. Group B also showed increased lymphocyte counts, further indicating immunomodulatory effects.

**Conclusion:**

CA exhibits considerable therapeutic potential in managing SCM, especially when combined with tylosin. Its antioxidative and immunomodulatory effects may enhance udder health and milk quality while reducing reliance on antibiotics. Future large-scale studies are warranted to confirm these findings and explore CA’s integration into sustainable mastitis management strategies.

## INTRODUCTION

Mastitis is a serious condition in dairy animals that adversely affect both milk quality and production levels [1]. Subclinical mastitis (SCM), unlike its clinical form, does not present visible symptoms or gross alterations in milk, making it detectable only through indirect diagnostic tools such as the California mastitis test (CMT), total somatic cell count (SCC), milk electrical conductivity (EC), and microbial culture. Its prevalence is estimated to be 40–50 times higher than clinical mastitis, which is more readily identifiable, thereby underscoring the need for timely intervention in the dairy sector [2]. At present, antibiotics are the cornerstone of SCM control. However, widespread antibiotic misuse has led to a global increase in antimicrobial-resistant microorganisms, while antibiotic residues in milk pose significant health threats to consumers through the accumulation of resistant bacterial strains [3].

In response to these challenges, interest in non-antibiotic alternatives, particularly herbal remedies, is growing. *Piper nigrum*, recognized for its antimicrobial, antioxidant, analgesic, and anti-inflammatory properties, has been traditionally employed in the treatment of mastitis in cattle within indigenous veterinary systems [4]. The use of clarified butter is known to enhance the therapeutic efficacy of herbs, and it is commonly combined with *P. nigru*m in ethnoveterinary formulations [5].

In a similar context, the current study investigates *Centratherum anthelminticum* (CA), commonly referred to as “Kaali Jeeri,” owing to its reported antimicrobial, anti-inflammatory, and antioxidant properties [6]. This herb offers a promising natural alternative to antibiotics for mastitis management, potentially addressing the issue of antibiotic resistance. Therefore, the study aimed to assess the therapeutic efficacy of CA in treating SCM in lactating cows and its influence on milk quality. The herb’s active constituents, such as terpenoids and phenolic compounds, are believed to exert biological effects by disrupting bacterial membranes and modulating inflammatory responses [7].

Despite the high prevalence of SCM and its detrimental impact on milk quality and udder health, the current therapeutic strategies remain largely dependent on antibiotic treatments. While antibiotics such as tylosin have demonstrated efficacy in reducing bacterial load and inflammation, their prolonged and indiscriminate use has led to the emergence of multidrug-resistant pathogens and the presence of harmful residues in milk, posing serious public health concerns. Although several herbal formulations, including *P. nigru*m, have shown promise in traditional mastitis treatment, empirical data validating their efficacy through controlled clinical studies remain limited. Furthermore, there is a scarcity of comparative investigations evaluating the standalone and combined effects of phytotherapeutics and conventional antibiotics on biochemical, hematological, and oxidative parameters in SCM-affected cattle. Notably, CA (commonly known as Kaali Jeeri), a plant with documented antimicrobial, anti-inflammatory, and antioxidant activities, has not been extensively studied in the context of mastitis. Existing literature lacks robust *in vivo* data on its therapeutic potential, optimal dosing, and mechanistic effects in lactating dairy cows suffering from SCM. This gap underscores the need for alternative, safe, and sustainable mastitis control strategies supported by evidence-based veterinary research.

This study aims to evaluate the therapeutic efficacy of CA in the treatment of SCM in lactating cattle. Specifically, it investigates the effects of CA, administered alone and in combination with tylosin, on milk biochemical composition, oxidative stress indices (total antioxidant capacity [TAC] and total oxidative stress [TOS]), and hematological parameters. The study also seeks to determine whether the integration of this herbal treatment can enhance milk quality, modulate systemic health markers, and provide a viable alternative or adjunct to antibiotic therapy. Through this investigation, the study endeavors to contribute to the development of plant-based, residue-free therapeutics for the sustainable management of mastitis in dairy herds.

## MATERIALS AND METHODS

### Ethical approval

This study was ethically approved by the Ethical Review Committee of the Faculty of Veterinary Science, Department of Clinical Medicine and Surgery, University of Agriculture, Faisalabad, Pakistan (Approval No. FVS-2024-MPHIL-CMS-086).

### Study period and location

The study was conducted from June 2024 to November 2024. Samples were collected from a private dairy farm located in Faisalabad, Pakistan and then transported to the Translational Medicine Laboratory at the University of Agriculture, Faisalabad for further analysis.

### Sample collection

A total of 15 milk samples were collected from lactating cattle suspected of having SCM. These samples were properly preserved and transported to the Translational Medicine Laboratory at the University of Agriculture, Faisalabad. The presence of SCM was confirmed through diagnostic procedures, including the CMT and SCC analysis [8].

In addition, blood samples were aseptically drawn from the jugular vein of CMT-positive cattle both before and after treatment. The blood samples were collected in sterile ethylenediaminetetraacetic acid vacutainers and transported under appropriate conditions for hematological analysis. The selected CMT-positive cows were randomly assigned to three treatment groups. All treatments were administered twice daily for 7 days, as detailed in Table 1.

**Table 1:** Tabulated representation of groups and their respective therapeutic treatments.

Animals group	Number of animals	Treatment duration	Treatment	Route and dosage
Experiment Group A (control)	5	7 days	Standard treatment with antibiotic (Tylosin)	Intramuscular (IM) route 18 mg/kg
Experiment Group B	5	7 days	*Centratherum Anthelminticum*	Orally, 120 g
Group C: Experimental	5	7 days	*Centratherum Anthelmintic* and Antibiotic (Tylosin)	Oral dose of 120 g and antibiotic IM 18 mg/kg

### Biochemical analysis of milk

Milk samples collected from affected cattle were analyzed for multiple biochemical parameters, including pH, lactose, protein, fat, solids-not-fat (SNF), TAC, and TOS. The milk pH was measured both before and after treatment using a Lactoscan milk analyzer to detect any deviations associated with spoilage or mastitis. The Lactoscan was also used to determine lactose, protein, fat, and SNF concentrations, which are indicative of milk compositional changes resulting from SCM [9].

TAC and TOS were assessed using a spectrophotometer to evaluate the oxidative balance within the milk. TAC reflects the total antioxidant potential, while TOS quantifies the oxidative load caused by reactive oxygen species [10]. These measurements provided insight into the oxidative status and biochemical disruptions associated with mastitis.

### Hematological analysis

Hematological evaluations were conducted using a fully automated hematology analyzer. The parameters assessed included hemoglobin (Hb), red blood cell (RBC) count, packed cell volume (PCV), and total leukocyte count, to identify variations indicative of systemic responses to SCM [11].

### Statistical analysis

All statistical analyses were performed using the SPSS software version 26 (IBM Corp., NY, USA). Data were analyzed using a one-way analysis of variance, and mean comparisons were conducted using the t-test to determine the significance of differences among treatment groups.

## RESULTS

### Biochemical analysis of milk

Milk samples from cattle diagnosed with SCM were subjected to biochemical evaluation to assess the efficacy of treatment. In Group B, which received CA, a significant reduction in SCC was observed, suggesting decreased mammary inflammation and improved udder health, with outcomes comparable to or exceeding those of the antibiotic-treated Group A. Group C, receiving both CA and tylosin, demonstrated statistically significant post-treatment increases in milk quality parameters, particularly lactose and SNF, indicating a synergistic effect of the combined treatment.

Although the improvements in Group B were less pronounced than in Group C, the results still reinforced the therapeutic potential of the herbal remedy. pH and EC values trended toward normalization across all groups, aligning with the mitigation of inflammation. Group B exhibited the most notable antioxidative effect, as indicated by a marked increase in TAC and reduction in TOS compared to Group A. While Group C also demonstrated improvements in oxidative balance, these were comparatively moderate.

### Hematological parameters and milk quality changes

Hematological parameters, including RBC count, white blood cell (WBC) count, and PCV remained within physiological limits in all groups. Slight, though non-significant, improvements were noted in Group C, suggesting favorable systemic responses to treatment.

### Comparative observations in Groups A and B

**Figure 1:**
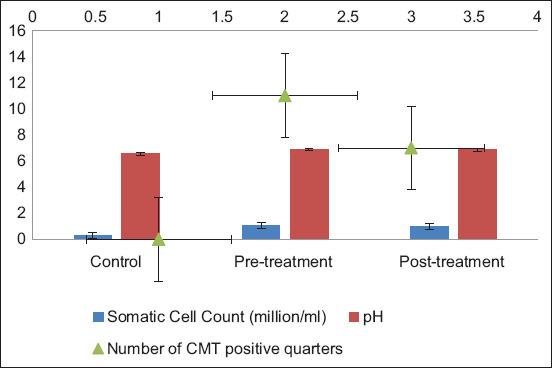
Group A. Comparison of the pre-treatment, post-treatment, and control groups.

In Group A, post-treatment reductions in SCC (1.00 ± 0.139 million/mL) and CMT-positive quarters (7) were recorded, compared with higher pre-treatment values (1.06 ± 0.126 million/mL; 11 quarters) (Table 2). Elevated milk pH levels (6.87–6.89) persisted, consistent with SCM pathology. These findings align with previous reports by McDougall *et al*. [12] on SCC and pH alterations during mastitis therapies. Figure 1 illustrates these changes in SCC, pH, and CMT-positive quarters across control, pre-treatment, and post-treatment groups.

**Table 2:** Pre-treatment and post-treatment variations in Group A.

Parameters	Control	Group A	

		Pre-treatment	Post-treatment
Somatic cell count (million/mL)	0.33^a^ ± 0.014	1.06^b^ ± 0.126	1.00^b^ ± 0.13
pH	6.57^a^ ± 0.018	6.89^b^ ± 0.04	6.87^b^ ± 0.03
Number of positive quarters of CMT	0	11	7

Significant differences (p < 0.05) exist between values with various superscripts for each individual parameter across rows. CMT=California mastitis test

**Figure 2:**
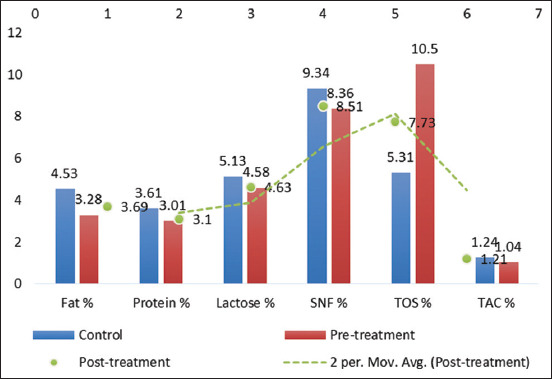
Evaluation of milk quality parameters in Group A before and after treatment.

Post-treatment in Group A also resulted in improved milk composition: Fat (3.69% ± 0.07%), protein (3.10% ± 0.07%), lactose (4.63% ± 0.06%), and SNF (8.51% ± 0.09%) all increased compared to the control. Concurrently, TOS levels declined (7.73% ± 0.09%) and TAC rose (1.21% ± 0.07%), indicating a reduction in oxidative stress and an improvement in milk quality [13]. These compositional changes are visualized in Figure 2, with trends indicating normalization following treatment.

**Figure 3:**
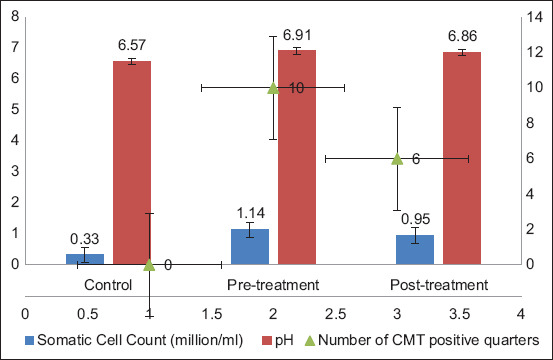
Parameter variations between pre-treatment and post-treatment in Group B.

In group B, the subclinical mastitis significantly increased SCC (0.33 ± 0.014 to 1.14 ± 0.185 million/mL) and milk pH (6.57 ± 0.018 to 6.91 ± 0.036), with a reduction in post-treatment (0.95 ± 0.178 million/mL and 6.86 ± 0.030, respectively). CMT-positive quarters decreased from 10 to 6 after treatment, indicating significant therapeutic efficacy. Figure 3 compares somatic cell count (million/mL), pH, and the number of CMT-positive quarters in control, pre-treatment, and post-treatment groups. Post-treatment results show a significant reduction in somatic cell count and the number of CMT-positive quarters (p < 0.05), indicating improved udder health, while pH levels remain non-significantly altered (p > 0.05).

**Figure 4:**
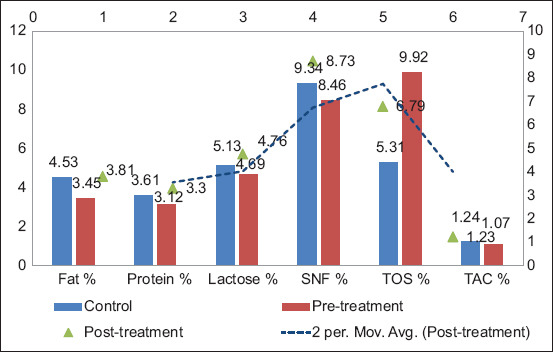
Diffusion of pre- and post-treatment milk quality parameters in Group B.

In Group B, SCM led to notable declines in milk component pre-treatment: Fat dropped from 4.53% ± 0.16% to 3.45% ± 0.17%, protein from 3.61% ± 0.05% to 3.12% ± 0.08%, lactose from 5.13% ± 0.04% to 4.70% ± 0.05%, and SNF from 9.34% ± 0.074% to 8.46% ± 0.111%. Post-treatment, only partial recovery was observed: Fat (3.81% ± 0.195%), protein (3.30% ± 0.095%), and lactose (4.76% ± 0.05%). TOS spiked from 5.31% ± 0.072% to 9.92% ± 0.541% pre-treatment, then fell to 6.79% ± 0.07% post-treatment, while TAC, slightly reduced pre-treatment, returned to baseline level (1.23% ± 0.08%) post-treatment [14]. These findings are illustrated in Figure 4.

### Hematological variations pre- and post-treatment

**Figure 5:**
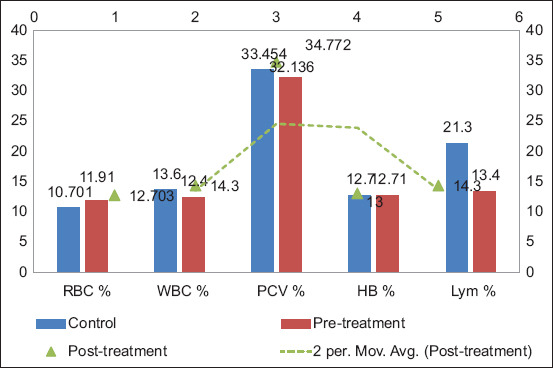
Hematological parameters before and after treatment in Group A.

Group A exhibited hematological changes consistent with mastitis and its resolution. RBC levels increased from 10.70% ± 0.21% to 11.91% ± 0.98% pre-treatment and further to 12.70% ± 0.35% post-treatment. PCV improved from 32.14% ± 0.84% to 34.77% ± 1.00%, while WBC count increased slightly from 12.40% ± 3.70% to 14.30% ± 6.30% post-treatment. Figure 5 illustrates these hematological changes, showing decreased values during infection and improvement post-treatment. Lymphocyte percentages declined during pre-treatment and stabilized afterward, with a moving average trend line confirming the consistency of post-treatment values [15].

**Figure 6:**
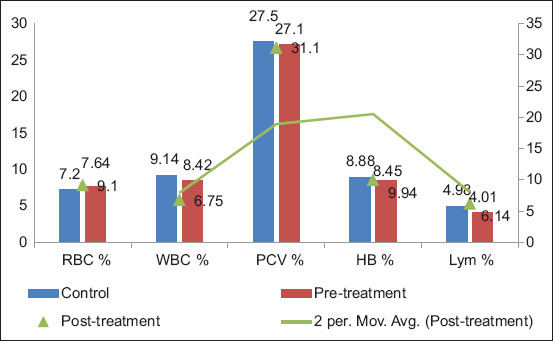
Evaluation of pre- and post-treatment hematological parameters in Group B.

Similarly, Group B demonstrated a slight decline in RBC (7.20% ± 0.35% to 7.64% ± 0.24%) and WBC (9.14% ± 0.40% to 8.42% ± 0.16%) pre-treatment, followed by post-treatment increases: RBC (9.10% ± 0.14%), Hb (from 8.88% ± 0.38% to 9.94% ± 0.25%), and PCV (27.5% ± 0.41% to 31.10% ± 0.42%). Lymphocyte percentages dropped from 4.98% ± 0.35% to 4.01% ± 0.18% before treatment and then rose to 6.14% ± 0.54% afterward [15]. Figure 6 presents these data using bar and line graphs, showing modest recovery in hematological indices and reinforcing the positive physiological effects of treatment.

## DISCUSSION

### Biochemical and hematological changes in SCM

The present study highlights the significant biochemical and hematological changes associated with SCM, evaluated using parameters such as the CMT, SCC, TAC, and TOS [16]. The elevated milk pH and EC in mastitic cows are consistent with the previous study by Pamparienė *et al*. [17] which reported similar correlations between these parameters and higher CMT and SCC scores in the mammary gland. This elevation is likely due to the infiltration of leukocytes and increased permeability of the mammary cell membrane, leading to the leakage of ions and salts [17].

Milk composition parameters, including protein, fat, lactose, and SNF percentages, were decreased across all treatment groups, imitating the effect of SCM on milk production [18]. This reduction may be due to the disruption of mammary gland function or damage to secretory cells caused by the ongoing infection. Such changes reflect pathogen-induced inflammatory responses that compromise the gland’s ability to maintain normal milk synthesis [19].

### Immune response and oxidative stress indicators

Hematological parameters showed increased leukocyte counts, particularly neutrophils, in mastitic milk, consistent with a previous study that highlighted neutrophils as key indicators of mammary infection. Similarly, increased RBC and WBC counts, PCV, and lymphocyte counts in blood samples from mastitic cattle suggested a systemic inflammatory response. The observed decreases in monocytes and macrophages in mastitic milk are consistent with the previous study by Sarvesha *et al*. [20]. Oxidative stress, as reflected by elevated TOS and reduced TAC levels in both milk and blood, was significantly higher in SCM cattle. These findings are consistent with the previous study by Wadhwani *et al*. [21], which shows that oxidative stress contributes to tissue damage and impaired mammary function in mastitis cows.

### Therapeutic role of herbal interventions

The results suggest that herbal remedies, particularly when combined with conventional antibiotics, may offer viable therapeutic options for dairy farmers seeking residue-free treatment alternatives. These results align with findings from previous studies by Amin *et al*. [22] and Okmen *et al*. [23], which compare different treatments for mastitis, including antibiotics and herbal approaches.

Despite statistically significant changes in milk quality parameters, such as SCC, fat, protein, lactose, and SNF percentages, these results highlight alterations in SCM. Significant improvements, like those observed in Group C (combination therapy), may have long-term positive effects on SCM in cattle. These trends, although significant in this study, suggest a potentially beneficial role for CA in enhancing udder health. Future studies with larger sample sizes may provide clearer insights and statistical significance, thereby confirming the observed trends. Thus, the current study suggested that CA has significant potential as an adjunctive treatment for SCM, recommending improvement in oxidative stress. These findings align with a previous study by Amin *et al*. [22] on herbal treatments for mastitis, which highlighted their antimicrobial and anti-inflammatory properties. However, CA differentiates itself through its antioxidant activity, which augments the overall therapeutic effect when combined with antibiotics, as shown by the improvements observed in TAC and TOS levels.

## CONCLUSION

This study demonstrated that CA possesses significant therapeutic potential in the management of SCM in lactating cattle. Cows treated with CA, either alone or in combination with tylosin, exhibited marked reductions in SCC and TOS, alongside increased TAC, indicating a clear improvement in udder health and oxidative balance. Notably, the combined treatment group (Group C) demonstrated statistically significant improvements in milk quality indicators, including lactose and SNF content, suggesting a synergistic effect of herbal and antibiotic therapy. From a practical standpoint, these findings offer a promising alternative or adjunct to conventional antibiotic therapy, aligning with the urgent need for antimicrobial stewardship and residue-free treatment strategies in dairy production. The enhanced oxidative profile and partial restoration of milk composition further highlight the physiological benefits of CA in improving both animal welfare and milk quality.

A key strength of this study lies in its dual assessment of both biochemical and hematological parameters, providing a comprehensive evaluation of the systemic and local effects of treatment. Besides, the study introduces CA as a novel, evidence-based herbal candidate for the treatment of bovine mastitis.

However, the current study is limited by its small sample size and short treatment duration, which may restrict the generalizability and long-term extrapolation of the findings. In addition, the specific active constituents and their pharmacokinetics remain unexplored, underscoring the need for further mechanistic studies. Future research should aim to validate these findings through larger, multi-site trials; investigate optimal dosing regimens; and assess the molecular mechanisms underlying the observed effects. Longitudinal studies assessing recurrence rates and production economic post-treatment will further strengthen the case for field-level adoption.

In conclusion, CA emerges as a scientifically supported phytotherapeutic agent with anti-inflammatory, antimicrobial, and antioxidant properties, offering a sustainable and effective strategy for managing SCM in dairy cattle. Its integration into veterinary protocols could contribute meaningfully to responsible antimicrobial use and improved herd productivity.

## DATA AVAILABILITY

All data generated or analyzed during this study are included in the manuscript.

## AUTHORS’ CONTRIBUTIONS

MA and FD: Conceived, designed, and coordinated the study. MA: Collected the animal samples and drafted the manuscript. MT, MU, and SS: Performed statistical analysis and interpreted the results. SS: Reviewed and edited the manuscript. E1A, PDRE, and MS: Project administration, visualization, and data curation. All authors have read and approved the final manuscript.
